# Analysis of Differential miRNA Expression in the Duodenum of *Escherichia coli* F18-Sensitive and -Resistant Weaned Piglets

**DOI:** 10.1371/journal.pone.0043741

**Published:** 2012-08-24

**Authors:** Lan Ye, Xianmin Su, Zhengchang Wu, Xianrui Zheng, Jin Wang, Chen Zi, Guoqiang Zhu, Shenglong Wu, Wenbin Bao

**Affiliations:** 1 Key Laboratory for Animal Genetics, Breeding, Reproduction and Molecular Design of Jiangsu Province, College of Animal Science and Technology, Yangzhou University, Yangzhou, Jiangsu Province, China; 2 College of Veterinary Medicine, Yangzhou University, Yangzhou, Jiangsu Province, China; Centre National de la Recherche Scientifique, Aix-Marseille Université, France

## Abstract

Small RNA duodenal libraries were constructed for *Escherichia coli* F18-sensitive and -resistant weaned piglets in full-sib pair groups and sequenced using Illumina Solexa high-throughput sequencing technology. The identification of differentially expressed miRNAs provides the basis for improved database information on pig miRNAs, understanding the genetic basics of differences in resistance to *E. coli* F18 between local Chinese and exotic pig breeds, and finding new resistance markers for *E. coli* F18 infection. The duodenum of all individuals contained more than 90% of known swine miRNAs. A total of 58 differentially expressing miRNAs were identified, of which 46 were increased and 12 were decreased in *E. coli* F18-sensitive pigs. Of miRNAs with increased expression, ssc-miR-143 was most highly expressed, followed by ssc-let-7f, ssc-miR-192, and ssc-miR-21. We identified a total of 2036 intersection target genes by comparing TargetScan data and previous gene expression profile results. Gene ontology and pathway analysis of intersection genes showed that differentially expressed miRNAs were mainly involved in the immune response and transcriptional regulation. Combining information on differential miRNA expression and their regulatory relationships with transcription factors, identified 12 candidate miRNA disease markers, including 11 miRNAs with increased expression, ssc-miR-143, ssc-let-7f, ssc-miR-30e, ssc-miR-148a, ssc-miR-148b, ssc-miR-181a, ssc-miR-192, ssc-miR-27b, ssc-miR-15b, ssc-miR-21, and ssc-miR-215, and one with decreased expression, ssc-miR-152. Quantitative real-time PCR analysis of candidate miRNA expression in a larger cohort of *E coli* F18-sensitive and -resistant animals confirmed the high-throughput sequencing results.

## Introduction

MiRNAs are endogenous 21–24 nucleotide non-coding RNAs that regulate gene expression in eukaryotes [1], [2]. Mature miRNAs are not directly produced by gene transcription, but are processed from long primary miRNA (pri-miRNA) precursors. Recent reports indicate that miRNAs can not only combine with the 3′UTR of target mRNAs but may also bind to sites in the coding region and 5′UTR to regulate genes involved in development, virus defense, cell proliferation, apoptosis, and fat metabolism [3], [4].

Studies into miRNA function have mainly focused on a variety of human diseases, particularly cancer, and mainly relate to the use of miRNAs as disease biomarkers and for monitoring drug efficacy. In another important human disease, diabetes, the study by Melkman *et al.* [5] into the effects of miRNAs on insulin synthesis revealed that knocking out miR-24, miR-26, miR-182, or miR-148 reduced the transcriptional activity of the insulin gene promoter, thereby reducing the level of insulin mRNA. Reiner and Willems [6] reported that biomarkers for the early diagnosis of human diseases are rapidly being developed. In the veterinary field, their ability to regulate gene expression and their stability make miRNAs useful tools to guide breeding programs for disease resistance in many types of livestock, including pigs.

Although there are currently 1043 human miRNAs in the miRBase (v18) database, only about 250 pig miRNAs have been identified so far. Initially, miRNAs in livestock such as cattle and pigs were discovered using homology searches. However, after the high-throughput detection platform was developed, high-density small RNA expression profiles could be rapidly identified using gene chips, although these were limited to the detection of known miRNAs. In recent years, the emergence of Solexa and 454 high-throughput sequencing technologies have enabled the direct sequencing of miRNAs and thus the discovery new miRNAs. Xie *et al.* [7] constructed a small RNA cDNA library for 16 tissues of different pig breeds, conducted deep sequencing on the miRNA transcriptome, and obtained 437 conserved and 86 predicted porcine miRNA sequences. Following this, Xie *et al.* customized gene chips and identified 58 miRNAs differentially expressed in native Chinese Tongcheng pigs and the Large White exotic breed that contain different lean meat percentages. Likewise, Li *et al.* [8] constructed small RNA cDNA libraries of pig tissues at different growth stages from the fetal period to adulthood, and sequenced these using the Genome Analyzer GA-I.

Our main research focus is diarrhea and edema disease of weaned pigs caused by *E. coli* F18. Previous studies have shown that *E. coli* F18 cells combine with the intestinal epithelial cell receptor of piglets through surface fimbriae, and then multiply and produce toxins, leading to piglet disease. Vogeli *et al.* [9] found that *FUT1* gene and the epithelial cell receptor gene were closely linked, and that a G to A mutation at locus M307 of the *FUT1* gene that alters the structure of the receptor could be used as a genetic marker for screening. However, no AA genotype has been detected following screening of dozens of local breeds in China [10]. Thus, the *FUT1* gene is not a suitable genetic marker for the selection of *E. coli* F18-resistant local Chinese pig breeds. It therefore seems that exotic breeds and Chinese local pig breeds have different resistance mechanisms to *E. coli* F18 infection.

We previously used gene chips to screen for differential gene expression in eight full-sib pair groups of *E. coli* F18-sensitive and -resistant Sutai pigs bred under the same conditions [11]. We have now constructed small RNA duodenal libraries of individual *E. coli* F18-sensitive and -resistant weaned piglets in full-sib pair groups and analyzed these by Illumina Solexa high-throughput sequencing to identify differentially expressed miRNAs. This provides improved database information on pig miRNAs, better understanding of the genetic basis of *E. coli* F18 resistance in local Chinese and exotic pig breeds, and lays new foundation for identifing new markers in *E. coli* F18 resistance.

## Materials and Methods

### 1. Animals

The Sutai pig, a new hybrid between the Duroc and Meishan breeds, was from the Center of Sutai Pig Breeding. Based on polymorphism at the M307 locus of the *FUT1* gene, *E. coli* F18-resistant and -sensitive populations have been established after several years of continuous selection and breeding [12]. *E. coli* F18-resistant and -sensitive Sutai pig populations were bred and grown under the same conditions. Here, part of the samples used in mciroarray analyses were also conducted for high-throughput sequencing. In previous mciroarray experiment, eight full-sib families were selected for *FUT1* genotype analysis with all piglets showing no symptoms of diarrhea and edema disease. Around the time of weaning, when 28-day-old piglets are most susceptible to *E. coli* F18, one resistant (AA genotype) and one sensitive (GG genotype) pair of full-sibs from the same family, with similar birth weights, weaning weights, and body sizes were assessed. The V-type secretion system was used to assay for functional adhesin and a receptor-binding assay was used to analyze and verify *E. coli* F18 resistance and susceptibility in individuals of the paired group, see detailed steps in our published paper [11]. Following analysis of all piglets from the eight families, four pairs of GG- and AA-type full-sib individuals and four pairs of AG- and AA-type full-sib individuals were selected as the paired experimental group for mciroarray analyses. After slaughter, duodenal tissues were collected, snap frozen in liquid nitrogen, and stored at −70°C. In this study, three independent pairs of GG and AA full-sibling individuals were selected for high-throughput sequencing.

All the animals included in the experiment were conducted in the Animal Hospital of Yangzhou University according to the regulation for the Administration of Affairs Concerning Experimental Animals (Ministry of Science and Technology, China, revised in June 2004) and approved by experimental animal using permit with No. SYXK(Su)2007-005.

### 2. Preparation of Small RNA Libraries and DNA Sequencing

In this study, high-throughput sequencing was conducted on independent samples from each individual animal. Total RNA was extracted from duodenal tissues with TRIzol (Invitrogen, Gaithersburg, MD, USA) using the one-step method, according to the manufacturer’s instructions. After purification, RNA concentration was analyzed using a Nanodrop ND-1000 (Nanodrop Technologies, Wilmington, DE) and quality testing was conducted using an Agilent 2100 BioAnalyzer (Agilent Technologies, Palo Alto, CA). Small RNA was purified from total RNA by polyacrylamide gel electrophoresis (PAGE) to enrich molecules in the range of 16–30 nt, and then 3′ and 5′ linker sequences were attached, and SuperScript II reverse transcriptase was used to synthesize cDNA. PCR amplification was conducted using the following procedure: 98°C for 30 s; 12 cycles of 98°C for 10 s, 60°C for 30 s, and 72°C for 15 s; and 72°C for 10 min. Solexa sequencing was conducted after the purification test of amplified cDNA.

### 3. Analysis of Sequence Data

Original data image files were transformed into sequence files using Illumina CASAVA software and then converted into a FASTQ file that was analyzed sing CLC Genomics Workbench 4.01 software (http://www.clcbio.com/). Sequence data was pruned to exclude low quality sequences, ambiguous bases, and connecting sequences, leaving 15–55 base sequences. All validated sequences were obtained for further analysis. Non-annotated reads were mapped to porcine gene groups and the sequence with overlap over 100 bp (might be repeated sequences) were excluded. Regions with more than five sequences (with a distance between two sequences of less than 20 bases) were selected as seed regions and extended 100 bases on either side. The resulting sequence was analyzed by RNAfold and miRDeep to determine the loop structure representing the novel miRNA and predict the mature miRNA.

### 4. Identification of miRNAs Differentially Expressed in *E. Coli* F18-sensitive and-resistant Pigs

After sequencing, the random variance model corrected t-test (RVM-T test) was used to calculate the significance level (*P*-value) of miRNA expression and the false positive rate (FDR), and miRNAs differentially expressed in *E. coli* F18-sensitive and-resistant pigs were identified. All differentially expressed miRNAs were compared with the Sanger database to determine the specific location of pri-miRNAs in the porcine genome and the UCSC Genome database was used to identify pri-miRNA sequences. The presence of transcription factor binding sequences in pri-miRNA regulatory regions was used to indicate transcription factors that may regulate their transcription. This information was used to construct a regulatory network of transcription factors and miRNA molecules.

### 5. Prediction and Analysis of miRNA Target Genes

As there is no appropriate database or methods for predicting pig miRNA target genes, the human orthologs of differentially expressed porcine miRNA sequences were used to identify potential target genes by searching the TargetScan database. All potential target genes were screened alongside differentially expressed genes on an expression profile chip [11]. Functional annotation, classification, and metabolism pathway analysis were conducted on selected target genes using the GenBank, GO, and KEGG databases.

### 6. Graph Theory: Network Analysis

We used Jemboss software to identify possible interactions between differentially expressed pri-microRNAs and transcription factors. In addition, JAVA and Cityscape 2.6.0 software were used to construct regulatory miR – GO and miRNA – target gene networks based on regulatory interactions between differentially expressed miRNAs and target GO genes. Many nodes exist in each network, and each node can represent a gene, a GO or something else. “Degree" in the figure represents the number of interactions between a single node and the surrounding nodes in the network; the higher the “degree", the more interactions there are between a node and the surrounding nodes.

### 7. Quantitation of Target miRNAs in *E Coli* F18-sensitive and -resistant Groups

Target miRNAs were identified in five *E. coli* F18-sensitive and five *E. coli* F18-resistant 28-day-old Sutai piglets classified by adhesin testing using the quantitative TaqMan miRNA Assay Kit (ABI, California, USA), with U6 snRNA as the internal reference.

For cDNA synthesis, a 15 µL reaction mixture contained 1.5 µL 10× Reverse Transcription Buffer, 1 µL MultiScribe Reverse Transcriptase (50 U/µL), 0.15 µL dNTPs containing dTTP (100 mmol/L), 3.0 µL 5× RT Primer, 0.19 µL RNase Inhibitor (20 U/µL), and 5 µL total RNA, and reaction conditions were 16°C for 30 min, 42°C for 30 min, and 85°C for 5 min. The quantitative real-time PCR (qRT-PCR) reaction mixture (20 µL) contained 1.33 µL cDNA, 1 µL TaqMan Small RNA Assay (20×), and 10 µL TaqMan Universal PCR Master Mix II (2×), and cycling conditions were 50°C for 2 min; and 40 cycles of 95°C for 10 min, 95°C for 15 s, and 60°C for 60 s. Solubility curves were analyzed after amplification. Three experiments were done for each sample and the mean value is presented. Relative quantification results were analyzed using the 2^−ΔΔCt^ method, and significant differences in expression between resistant and sensitive individuals were determined using a t-test.

Data discussed in this publication have been deposited in the NCBI Gene Expression Omnibus and are accessible through GEO Series accession number GSE32527.

## Results

### 1. Identification of miRNAs Differentially Expressed in the Duodenum of *E. Coli* F18-sensitive and -resistant Pigs

Whole-genome sequencing of all individuals in the *E. coli* F18-sensitive and -resistant groups generated more than twenty million sequences for each individual. All reads were processed and the results are shown in Table S1. We identified between several thousand and over ten thousand unannotated miRNAs in the duodenum of each individual. The free energy value of each sequence was used to determine the structural stability of putative novel miRNAs. Table S2 lists potential new miRNA molecules from one individual in each comparison group.

### 2. Identification and Genome Location of miRNAs Differentially Expressed in *E. Coli* F18-sensitive and -resistant Groups

A total of 58 miRNAs were differentially expressed in the duodenum of *E. coli* F18-sensitive and-resistant pigs, of which 46 were increased and 12 were decreased in sensitive animals. MiRNAs with a mean value >10000 by sequence counting (i.e., expression) included ssc-miR-143, ssc-let-7f, ssc-miR-192, ssc-miR-21, ssc-miR-215 and ssc-miR-378. Analysis of pri-miRNA sequences identified two miR-378 precursors: ssc-miR-378-1 and ssc-miR-378-2. The specific genome locations of the precursor sequences of differentially expressed miRNAs were determined using the Sanger database. A total of 14 pri-miRNA sequences were located within gene sequences and 45 were located in intergenic regions. However, it should be noted that a single miRNA, for example such as ssc-miR-103-2, ssc-miR-27b, ssc-miR-23b, and ssc-miR-125b-1, may have multiple precursor targeting regions in the genome. Table S3 lists all differentially expressed miRNAs and pri-miRNAs corresponding to known human miRNAs.

### 3. Transcription Factor – gene Network

**Figure 1:**
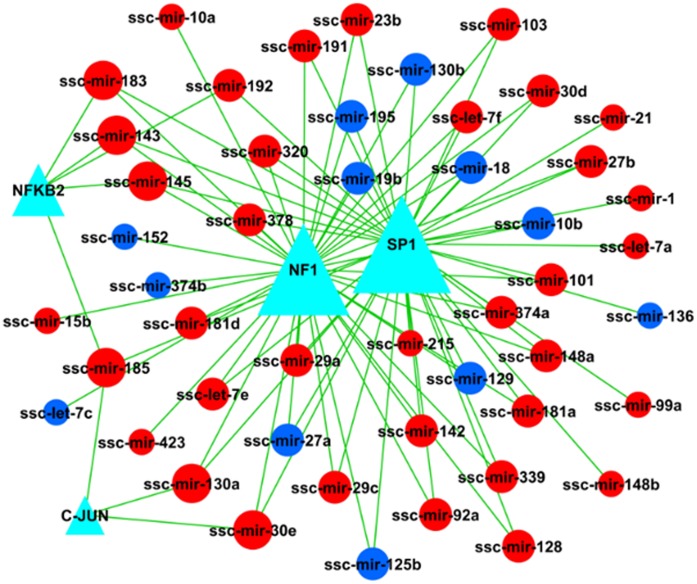
Network of transcription factors regulating differential miRNAs. Note: Dark blue triangles represent transcription factors, circles represent miRNA; straight lines represent the regulatory relationship between transcription factors and miRNA. Triangle size represents the number of transcription factors regulating the miRNA, circle size represents the number of miRNAs regulated by transcription factors. Red represents increased miRNA expression, blue represents decreased miRNA expression.

By matching miRNA precursor and transcription factor recognition sites, transcription factors regulating pri-miRNA expression were identified and a regulatory network of transcription factor and miRNAs was constructed (Figure 1). Based on the number of potential transcription factor interactions with single pri-miRNAs, key transcription factors regulating differential miRNA expression were identified. Table S4 shows the attributes of all transcription factor nodes in Figure 1. A single miRNA precursor in the network was regulated by three transcription factors at most, that is, the maximum degree is 3. The expression of all miRNAs with 3 degrees of regulation is increased miRNAs in sensitive pigs (Table S5).

### 4. Function Analysis of Intersection Genes between Differential miRNA Target Genes and Differential Expressed Genes

By comparing pig miRNA sequences with human miRNAs in the TargetScan database, 26232 porcine miRNA target genes with differential expression in *E. coli* F18-sensitive and -resistant pigs were identified (Table S6). These genes were compared with genes previously identified using expression file chips and 2036 corresponding relationships between miRNA target genes and genes with differential expression profiles were found (Table S7).

#### 4.1 MiRNA – gene ontology network analysis of intersection genes

Functional analysis was conducted on genes identified as both targets of differentially expressed miRNAs and those with differential expression profiles. As intersection genes were involved in more GO categories, significance was set at *p*<0.01. A total of 139 target genes corresponding to miRNAs with increased expression in sensitive animals gave results of functional significance (Table S8) and a 102 target genes corresponding to miRNAs with reduced expression in sensitive animals gave data of functional significance (Table S9). Target genes corresponding to these differentially expressed miRNAs were mainly involved in cell adhesion, positive regulation of transcription, positive regulation of apoptosis, and lipopolysaccharide response. Combined with biological functions that may be related to *E. coli* F18 infection, further screening was conducted on all target genes of miRNAs with increased or decreased expression in *E. coli* F18-sensitive pigs. A total of 53 significant GO categories were obtained and used to construct a miRNA – GO network of differentially expressed miRNAs. The main functions of key differential miRNAs and target genes regulated by these miRNAs were selected from samples of both groups (Figure S1). In addition, the graph theory method was used to define the regulation of differentially expressed miRNAs, GO categories in the network were assessed, and key differential miRNAs and GO categories were obtained (Table S10 and Table S11).

#### 4.2 Analysis of signaling pathways affecting expression of intersection genes

Signal transduction pathways associated with intersection genes corresponding to differentially expressed genes and target genes of differentially expressed miRNAs were next investigated. Screening criteria for defining significant pathways was *p*<0.05. Target genes corresponding to miRNAs with increased expression in sensitive animals were involved in 32 significant signal transduction pathways (Table S12), and those corresponding to miRNAs with decreased expression were involved in 28 significant signal transduction pathways (Table S13; LgP histograms are shown in Figures S2 and S3). Target genes corresponding to these miRNAs mainly participate in pathways linked to the immune response, but are not involved in pathways related to glycolipid synthesis and metabolism, as was found in the chip results.

**Figure 2:**
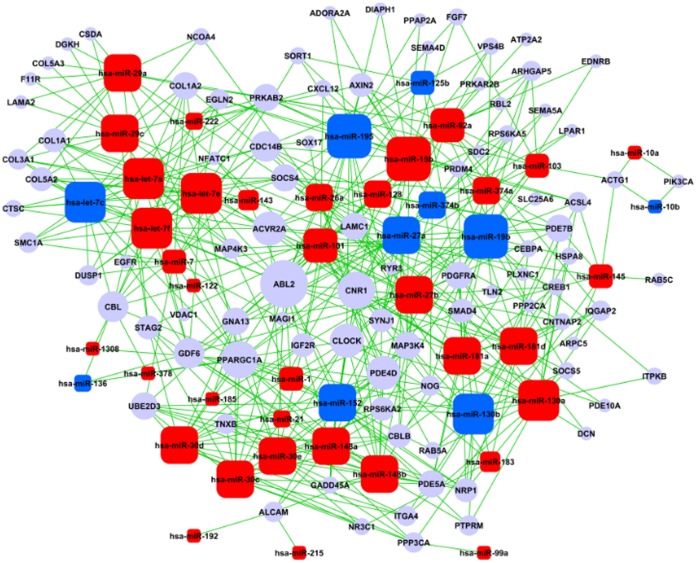
Regulatory network of differentially expressed miRNAs and intersection target genes associated with significant GO and pathway analyses. Note: Rectangles with rounded corners figure represent differentially expressed miRNAs (red – increased, blue – decreased); circles represent genes; lines represent regulatory relations between differentially expressed miRNAs and genes. Size of points is related to the degree of interaction.

#### 4.3 Construction of an overall regulatory network

Using the GO and pathway significance analysis of intersection genes, target genes associated with both types of analysis were obtained. Based on the functions of miRNAs and their target genes in both analyses, a regulatory miRNA – target gene network was constructed (Figure 2). In addition, the regulation status of miRNAs in the network was assessed using the graph theory method to identify key miRNAs involved in disease sensitivity (Table S14).

### 5. Identification of Differentially Expressed miRNA Candidates

Twelve miRNAs that were differentially expressed in *E. coli* F18-sensitive and -resistant pigs in all network analyses (Table S10, S11 and S14), involved in *E. coli* F18 pathogenesis and the immune response, and involved in regulating the lipopolysaccharide immune response were selected for further study. These included 11 with increased miRNA expression in *E. coli* F18-sensitive pigs, ssc-miR-143, ssc-let-7f, ssc-miR-30e, ssc-miR-148a, ssc-miR-148b, ssc-miR-181a, ssc-miR-192, ssc-miR-27b, ssc-miR-15b, ssc-miR-21, ssc-miR-215, and one with reduced miRNA expression, ssc-miR-152.

### 6. qRT-PCR Confirmation of Differential miRNA Expression

As no commercial TaqMan miRNA assay kit is available for ssc-miR-30e, ssc-miR-148b, and ssc-miR-181a, we conducted qRT-PCR validation of the remaining nine target miRNAs between *E coli* F18-sensitive and -resistant groups with an increased sample size. Quantitative results were consistent with our high-throughput sequencing results (Figure S4). As shown in Table 1, miR-215 and miR-192 expression was significantly different between *E coli* F18-sensitive and -resistant groups (*p*<0.01). Previous results obtained using microarray found five target genes corresponding to miR-215 (ALCAM, DLG5, FRMD4B, MIPOL1, and ZFHX3). However, only ALCM, associated with leukocyte adhesion, corresponded to miR-215 in the miRNA – gene network. Coincidentally, a comparison of the expression profile results and the miRNA – target gene network showed that target genes corresponding to miR-192 were identical to those of miR-215. In addition, the expression of miR-15b, miR-152, miR-143-5p, and let-7f were significantly different in the *E coli* F18-sensitive and -resistant groups (*p*<0.05). Target gene prediction for these miRNAs is shown in Table S7.

**Table 1:** qRT-PCR results of target miRNAs in *E coli* F18-sensitive and -resistant groups.

No.	miRNA	Sensitive group	Resistant group	*P*-value
1	ssc-mir-27b	3.319±1.161	2.565±1.253	0.219
2	ssc-mir-215	5.079±2.986^a^	2.463±1.510^b^	0.003
3	ssc-mir-21	4.833±1.884	3.281±1.734	0.083
4	ssc-mir-192	2.835±1.209^a^	1.730±0.562^b^	0.002
5	ssc-mir-15b	4.094±2.807^a^	2.275±0.900^b^	0.019
6	ssc-mir-148a	3.547±2.073	2.742±0.950	0.155
7	ssc-mir-143-5p	3.819±1.364^a^	2.062±1.347^b^	0.027
8	ssc-let-7f	8.250±2.607^a^	4.284±2.560^b^	0.030
9	ssc-mir-152	3.237±1.242^a^	5.495±1.446^b^	0.030

Note: Means with the different superscripts within the same line differ significantly (*P*<0.05).

## Discussion

Piglets are most susceptible to diarrhea and edema caused by *E. coli* F18 infection at weaning time, and this causes huge economic losses to the pig industry. European breeders have bred *E. coli* resistant Large White pigs using the *FUT1* gene M307 locus as a genetic marker. However, there are differences in the genetic basis of resistance to *E. coli* F18 infection between Chinese and foreign breeds. The Sutai pig has previously been used as a link between Chinese and foreign pig breeds, and differential gene expression in *E. coli* F18-sensitive and -resistant weaned piglet groups has been identified using gene chip technology [11]. In this study, sequencing of small RNAs from the duodenum of *E. coli* F18-sensitive and -resistant individuals was done using Illumina Solexa technology, and differentially expressed miRNAs were identified. We found that more than 90% of known swine miRNAs are expressed in duodenal tissues of all weaned piglets. In addition, a large number of small RNAs had sequences consistent with miRNA structure, indicating that physiological functions of the pig intestinal tract are regulated by miRNAs. As the swine and human miRNA sequences show a high degree of homology, alignment of porcine miRNAs with human gene bank sequences provides an effective way to identify porcine miRNA target genes in the absence of specific porcine miRNA target gene information.

There are relatively few reports of piglet intestinal miRNA sequencing. Sharbati *et al.* [13] conducted miRNA cDNA library sequencing on six different sections (duodenum, anterior segment of jejunum, posterior segment of jejunum, ileum, ascending colon, and transverse colon) of 31-day-old Euroc and Piétrain hybrid pig intestinal tissues, and found that miR-194 and miR-215 were highly expressed in the duodenum and posterior segment of the jejunum and that miR-19b, miR-23a, miR-24, and miR-30b expression was higher in the colon than in other sections of the intestinal tract. In this study, sequencing from the duodenum of individuals in *E. coli* F18-sensitive and -resistant groups identified 58 differentially expressed miRNAs, of which 46 were increased and 12 were decreased in the *E. coli* F18-sensitive group. As miRNAs can degrade target mRNA, we speculate that mRNA degradation may affect factors that protect against *E. coli* F18 infection the sensitive group, resulting in weakened resistance to disease. miRNAs overexpressed in *E. coli* F18-sensitive individuals include ssc-miR-143 (highest expression), ssc-let-7f, ssc-miR-143, ssc-miR-192, ssc-miR-21, ssc-miR-215, ssc-miR-378, ssc-miR-145, ssc-miR-26a, and ssc-miR-30e. Differences between our results and those of Soroush may be caused by different genetic background among different pig breeds. However, some highly expressed miRNAs in *E. coli* F18-sensitive group directly implies the great possibilities that piglet intestinal tract-related functions may be affected by them. Esau *et al.* [3] reported that the function of ssc-miR-143 is related to fat metabolism in mammals, and that increased ssc-miR-143 expression may promote fat cell differentiation. Therefore, the function of ssc-miR-143, which has the highest differential expression in the intestinal tract, may be related to a rapid growth phase in weaned piglets.

Many studies have shown tissue-specific miRNA expression in pigs. Reddy *et al.* [14] sequenced small RNA cDNA libraries of three pig tissues, heart, liver, and thymus, and identified tissue-specific expression of 120 pig miRNAs. Analysis of expression of these miRNAs in 14 pig tissues revealed that miR-499 and miR-208 were expressed mainly in the heart and miR-122 mainly in the liver. In addition, miR-1 and miR-133 had highest levels of expression in the heart, miR-181a and miR-142-3p in the thymus, miR-194 in the liver, and miR-143 in the stomach. miR-22, miR-26b, miR-29c, and miR-30c were widely expressed in various tissues. Jung *et al.* [15] conducted high-throughput sequencing of pig fibroblasts and identified 25 new miRNAs. A study on the expression profile of novel miRNAs in 11 pig tissues, including brain, liver, heart, muscle, colon, kidney, lung, spleen, stomach, cervix and fibroblasts, indicated that ssc-miR-15b, ssc-miR-23a, ssc-miR-23b, ssc-miR-24, ssc-miR-29a, ssc-miR-30b, ssc-miR-145, and ssc-miR-152 are expressed in all tissues; ssc-miR-210 is mainly expressed in the liver, spleen, and stomach; ssc-miR-34a and ssc-miR-130a are mainly expressed in the lungs; and ssc-miR-140 is highly expressed in the cervix. Consistent with these results, we found that miRNAs that are highly expressed in other tissues are also expressed in duodenum, but at lower levels. In line with previous studies, we next aim to determine the tissue-specific expression of miRNAs related to *E. coli* F18 infection.

miRNAs are known to regulate gene expression during development. However, miRNAs are also encoded by genes and regulated by transcription factors. We found that of the miRNAs identified in this study, six upregulated in *E. coli* F18-susceptible pigs, ssc-miR-30e, ssc-miR-185, ssc-miR-183, ssc-miR-145, ssc-miR-143, and ssc-miR-130a, are regulated by three different transcription factors. Therefore, these miRNAs may be the main regulators of target gene expression in thee duodenum and thus control the physiological function of the porcine intestine. As the porcine intestines investigated were infected by *E. coli* F18, the function of these six miRNAs may be most closely linked to *E. coli* pathogenesis.

Using homologous sequences in the human gene bank, we performed GO analysis on miRNAs and their target genes identified in our expression analysis and found that these genes are mainly involved in cell adhesion, transcriptional regulation, apoptosis regulation, and the response to lipopolysaccharides. In addition, pathway analysis suggested that these intersected genes are involved in multiple signaling pathways involved in the immune response, such as the Wnt, MAPK, cytokine, and T cell receptor signaling pathways. This suggests that the function of these miRNAs mainly relate to cell differentiation and the immune response, but not to porcine intestinal epithelial receptor synthesis pathway, previously identified by gene chip screening. Thus, production of the *E. coli* F18 adhesin receptor may not be directly regulated by miRNAs.

Our previous study indicated that the most common Chinese porcine *FUT1* M307 genotype is GG, i.e., the genotype expressing the receptor that facilitates *E. coli* F18 infection. However, Chinese pigs are known to be highly resistant to *E. coli* F18 infection. We therefore presume that differences in the immune system between individuals play an important role in resistance to pathogens. It is possible that Chinese pigs are inherently immune to *E. coli* F18. Interestingly, the functions of miRNAs identified in this study mainly related to the immune response and innate immunity.

This study used high-throughput sequencing to compare miRNA expression in pigs susceptible and resistant to *E. coli* F18 infection and identified 12 miRNAs with differential expression, including 11 upregulated in susceptible animals, ssc-miR-143, ssc-let-7f, ssc-miR-30e, ssc-miR-148a, ssc-miR-148b, ssc-miR-181a, ssc-miR-192, ssc-miR-27b, ssc-miR-15b, ssc-miR-21, ssc-miR-215, and one down-regulated, ssc-miR-152. qRT-PCR confirmed these results. Since little information is available on pig miRNA function, we used information on human homologs to understand the function of these miRNAs. Esau *et al.* [3] reported that miR-143 is involved in fat metabolism in mammals, and other studies have shown that miR-143 expression is linked to colon and prostate cancer [16], [17]. Let-7f inhibits IL-23 receptor expression in human CD4^+^ T cells [18]. In addition, miR-30e is activated by the beta-catenin/TCF4 pathway during intestinal cell differentiation [19]. Yue *et al.* found that miR-148a and miR-152 expression is reduced in gastrointestinal cancers compared with para-cancerous tissue [20]. Further, Li *et al.* observed that miR-181a expression is associated with T lymphocyte antigen sensitivity and TCR signaling. Neilon *et al.* reported that miR-181a is highly expressed during T lymphocyte maturation and that high levels of miR-181a expression improve B lymphocyte differentiation in mice [21]–[23]. Moreover, miR-148b expression is low in gastric cancers and suppresses cell differentiation through its tumor suppressor function [24]. Wang *et al.* investigated miRNA expression in murine lung tissue after LPS stimulation and found temporal changes in the expression of 12 miRNAs, including miR-27b, indicating a relationship between miR-27b expression and innate immunity [25]. Besides, during antigen-induced CD8^+^ T cell differentiation, miRNA expression was down-regulated compared with unstimulated T cells, and miR-15b is reported to play an important role in lymphocyte growth and function [26]. MiR-21 is widely expressed in mammals and is overexpressed in lung, breast, glioblastoma, stomach, pancreatic, liver, colon, and ovarian cancers, indicating an essential role in cancer development. Interestingly, one of the miR-21 target genes is the PTEN tumor suppressor [27]. In addition, both miR-192 and miR-215 are tumor suppressors and upregulation of miR-215 inhibits the differentiation of colon cancer stem cells [28], [29]. Therefore, the miRNAs identified in this study have important functions in intestinal disease and contribute to variations in immune function between individuals.

Our further studies will aim to investigate the function of candidate miRNAs in intestine epithelial cells by altering their expression and investigating the effect on the immune response to *E. coli* F18 infection by measuring levels of cytokine secretion and cell surface antigen expression. In addition, an ectopic target reporter gene could be developed to identify *E. coli* F18-resistant pigs for an improved pig-breeding program.

## Supporting Information

Figure S1 **Network of differential miRNAs regulating significant GO categories that may be related to** ***E.coli*** **F18 infection.** Note: Rectangles with rounded corners represent differentially expressed miRNAs (red – increased, blue – decreased in sensitive pigs); circles represent genes; and lines represent the regulatory interactions between miRNAs and genes. The size of the figure points is related to their degree.
(TIF)

Figure S2 **Significant pathway of increased miRNA target genes -(-LgP) histogram.**
(TIF)

Figure S3 **Significant pathway of decreased miRNA target genes -(-LgP) histogram.**
(TIF)

Figure S4 **Quantitation of miRNA targets in** ***E. coli*** **F18-sensitive and -resistant groups.l** Note: 1, ssc-miR-27b; 2, ssc-miR-215; 3, ssc-miR-21; 4, ssc-miR-192; 5, ssc-miR-15b; 6, ssc-miR-148a; 7, ssc-miR-143–5p; 8, ssc-let-7f; 9, ssc-miR-152.
(DOCX)

Table S1 **Comparison of sequencing results in** ***E. coli*** **F18 sensitive and resistant groups.**
(DOC)

Table S2 **New miRNAs predicted in** ***E. coli*** **F18-sensitive and –resistant groups.**
(XLS)

Table S3 **Differentially expressed miRNAs between** ***E. coli*** **F18-sensitive and -resistant groups.**
(DOC)

Table S4 **Node attributes of all transcription factors.**
(DOC)

Table S5 **miRNAs regulated by three different transcription factors.**
(DOC)

Table S6 **The correspondent target gene from human gene bank for different miRNAs (26232).**
(XLS)

Table S7 **The intersection gene between target gene and expression profiles.**
(XLS)

Table S8 **Function analysis of target genes for upregulated miRNAs (139).**
(XLS)

Table S9 **Functional analysis of target genes for downregulated miRNAs (102).**
(XLS)

Table S10 **The attribute relations between miRNAs in networks (degree >50) in** **Figure 1** **.**
(DOC)

Table S11 **Relationships between GO categories (degree >25) in** **Figure 1** **.**
(DOC)

Table S12 **Significant signaling pathways of gene targets of upregulated miRNAs.**
(XLS)

Table S13 **Significant signaling pathways of gene targets of downregulated miRNAs.**
(XLS)

Table S14 **Key miRNAs in network (degree >15).**
(DOC)
