# Supplementary figures and images for: Analysis of Differential miRNA Expression in the Duodenum of Escherichia coli F18-Sensitive and -Resistant Weaned Piglets
Source: PLoS One. 2012 Aug 24;7(8):e43741. doi: 10.1371/journal.pone.0043741 (PMC3427155; doi:10.1371/journal.pone.0043741)

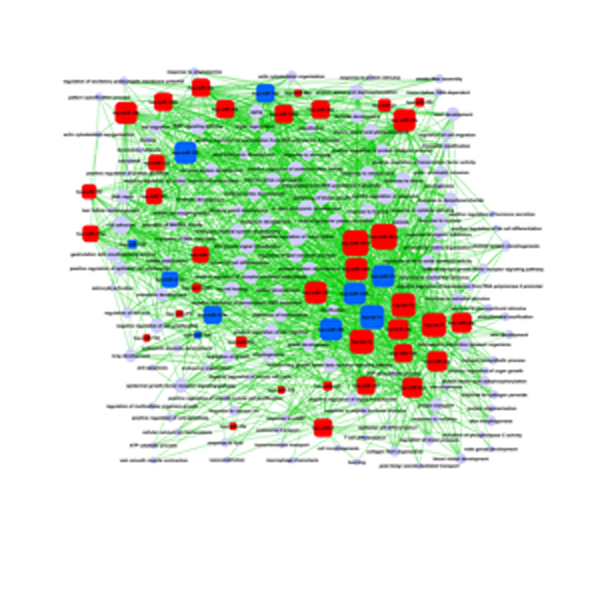

Supplement: Figure S1 — Network of differential miRNAs regulating significant GO categories that may be related to E.coli F18 infection. Note: Rectangles with rounded corners represent differentially expressed miRNAs (red – increased, blue – decreased in sensitive pigs); circles represent genes; and lines represent the regulatory interactions between miRNAs and genes. The size of the figure points is related to their degree. (TIF) [file pone.0043741.s001.tif]

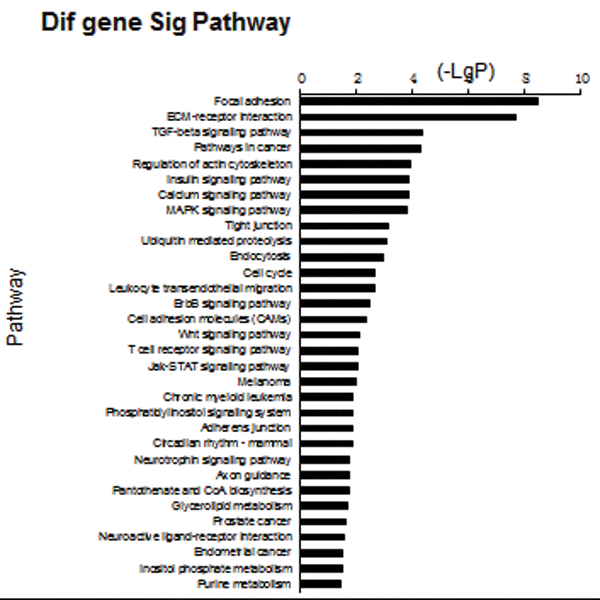

Supplement: Figure S2 — Significant pathway of increased miRNA target genes -(-LgP) histogram. (TIF) [file pone.0043741.s002.tif]

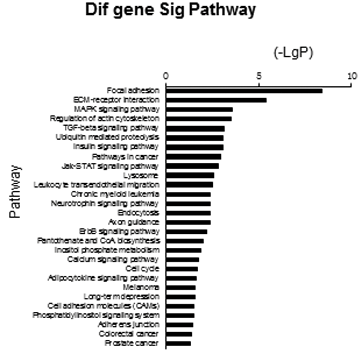

Supplement: Figure S3 — Significant pathway of decreased miRNA target genes -(-LgP) histogram. (TIF) [file pone.0043741.s003.tif]
