# Supplementary material for: Analysis of Differential miRNA Expression in the Duodenum of Escherichia coli F18-Sensitive and -Resistant Weaned Piglets
Source: PLoS One. 2012 Aug 24;7(8):e43741. doi: 10.1371/journal.pone.0043741 (PMC3427155; doi:10.1371/journal.pone.0043741)
Supplement: Figure S4 — Quantitation of miRNA targets in E. coli F18-sensitive and -resistant groups.l Note: 1, ssc-miR-27b; 2, ssc-miR-215; 3, ssc-miR-21; 4, ssc-miR-192; 5, ssc-miR-15b; 6, ssc-miR-148a; 7, ssc-miR-143–5p; 8, ssc-let-7f; 9, ssc-miR-152. (DOCX) [file pone.0043741.s004.docx]

**Figure S4 Quantitation of miRNA targets in *E. coli* F18-sensitive and -resistant groups**

Note: 1, ssc-miR-27b; 2, ssc-miR-215; 3, ssc-miR-21; 4, ssc-miR-192; 5, ssc-miR-15b; 6, ssc-miR-148a; 7, ssc-miR-143–5p; 8, ssc-let-7f; 9, ssc-miR-152.
