# Supplementary material for: Analysis of Differential miRNA Expression in the Duodenum of Escherichia coli F18-Sensitive and -Resistant Weaned Piglets
Source: PLoS One. 2012 Aug 24;7(8):e43741. doi: 10.1371/journal.pone.0043741 (PMC3427155; doi:10.1371/journal.pone.0043741)
Supplement: Table S1 — Comparison of sequencing results in E. coli F18 sensitive and resistant groups. (DOC) [file pone.0043741.s005.doc]

**Table S1. Comparison of sequencing results in *E. coli* F18 sensitive and resistant groups**

| Items | Average value in sensitive group | Average value in resistant group |
| --- | --- | --- |
| Number of reads | 23,297,324 | 24,700,374 |
| Number of trimmed reads | 12,712,423 | 15,043,548 |
| Average length of trimmed reads | 24.2 | 24.2 |
| Number of trimmed annotated reads | 4,526,417 | 5,633,471 |
| Number of small RNAs | 459,973 | 575,436 |
| Number of small annotated RNAs | 26,191 | 23,673 |
| Number of small annotated RNAs mapped to miRBase (*Sus Scrofa*) v15.0（175 in all） | 163 | 165 |
| Number of small annotated RNAs mapped to miRBase (*Homo Sapiens*) v15.0（940 in all） | 238 | 244 |
