# Supplementary material for: Analysis of Differential miRNA Expression in the Duodenum of Escherichia coli F18-Sensitive and -Resistant Weaned Piglets
Source: PLoS One. 2012 Aug 24;7(8):e43741. doi: 10.1371/journal.pone.0043741 (PMC3427155; doi:10.1371/journal.pone.0043741)
Supplement: Table S3 — Differentially expressed miRNAs between E. coli F18-sensitive and -resistant groups. (DOC) [file pone.0043741.s007.doc]

**Table S3 Differentially expressed miRNAs between *E. coli* F18-sensitive and -resistant groups**

| Feature ID  (Sus scrofa) | *Sus scrofa*  mature  miRNA | *Sus scrofa*  miRNA  precursor | Location of precursor | Average  value of  GG genotype | Average  value of  AA genotype | *P*-value | FDR | UP or  DOWN | Human mature  miRNA |
| --- | --- | --- | --- | --- | --- | --- | --- | --- | --- |
| mir-23b | ssc-let-23b | ssc-mir-23b | 10 | 1899 | 1863 | 0.000511 | 0.00118 | up | hsa-let-23b |
| let-7a | ssc-let-7a | ssc-let-7a | 9 | 749 | 877 | 0 | 0.001841 | up | hsa-let-7a |
| let-7e | ssc-let-7e | ssc-let-7e | 6 | 97 | 155 | 0 | 0.004947 | up | hsa-let-7e |
| let-7f | ssc-let-7f | ssc-let-7f | X | 34032 | 43894 | 0 | 0.000237 | up | hsa-let-7f |
| mir-1 | ssc-mir-1 | ssc-mir-1 | 17 | 108 | 194 | 0 | 0.004494 | up | hsa-mir-1 |
| mir-101–1 | ssc-mir-101 | ssc-mir-101–1 | 1 | 6262 | 4891 | 0 | 0.000663 | up | hsa-mir-101 |
| mir-101–2 | ssc-mir-101 | ssc-mir-101–2 | 6 | 5711 | 4401 | 0 | 0.000699 | up | hsa-mir-101 |
| mir-103–1 | ssc-mir-103 | ssc-mir-103–1 | 16 | 505 | 1021 | 0 | 0.001904 | up | hsa-mir-103 |
| mir-103–2 | ssc-mir-103 | ssc-mir-103–2 | 17 | 512 | 1026 | 0 | 0.001896 | up | hsa-mir-103 |
| mir-10a | ssc-mir-10a | ssc-mir-10a | 12 | 297 | 343 | 0.000444 | 0.003018 | up | hsa-mir-10a |
| mir-1308 | ssc-mir-1308 | ssc-mir-1308 | - | 1104 | 1551 | 0 | 0.00142 | up | has-mir-1308 |
| mir-130a | ssc-mir-130a | ssc-mir-130a | 2 | 57 | 116 | 0 | 0.006038 | up | hsa-mir-130a |
| mir-142 | ssc-mir-142 | ssc-mir-142 | 12 | 583 | 874 | 0 | 0.001951 | up | hsa-mir-142 |
| mir-143 | ssc-mir-143 | ssc-mir-143 | 2 | 36285 | 38490 | 0 | 0.000242 | up | hsa-mir-143 |
| mir-145 | ssc-mir-145 | ssc-mir-145 | 2 | 9221 | 9821 | 0 | 0.0005 | up | hsa-mir-145 |
| mir-148a | ssc-mir-148a | ssc-mir-148a | 18 | 5812 | 6907 | 0 | 0.000619 | up | hsa-mir-148a |
| mir-148b | ssc-mir-148b | ssc-mir-148b | 5 | 130 | 181 | 4.90E-05 | 0.004425 | up | hsa-mir-148b |
| mir-15b | ssc-mir-15b | ssc-mir-15b | 13 | 794 | 528 | 2.00E-06 | 0.002055 | up | hsa-mir-15b |
| mir-181a-1 | ssc-mir-181a | ssc-mir-181a-1 | 10 | 1697 | 1891 | 0 | 0.00121 | up | hsa-mir-181a |
| mir-181a-2 | ssc-mir-181a | ssc-mir-181a-2 | 1 | 1693 | 1890 | 0 | 0.001211 | up | hsa-mir-181a |
| mir-181d | ssc-mir-181d | ssc-mir-181d | 2 | 62 | 117 | 1.00E-06 | 0.00593 | up | hsa-mir-181d |
| mir-183 | ssc-mir-183 | ssc-mir-183 | 18 | 2223 | 3381 | 0 | 0.000955 | up | hsa-mir-183 |
| mir-185 | ssc-mir-185 | ssc-mir-185 | 14 | 69 | 99 | 0.001941 | 0.006133 | up | hsa-mir-185 |
| mir-191 | ssc-mir-191 | ssc-mir-191 | 13 | 6242 | 6909 | 0 | 0.000608 | up | hsa-mir-191 |
| mir-192 | ssc-mir-192 | ssc-mir-192 | 2 | 22675 | 18691 | 0 | 0.000331 | up | hsa-mir-192 |
| mir-21 | ssc-mir-21 | ssc-mir-21 | 12 | 13617 | 14872 | 0 | 0.000403 | up | hsa-mir-21 |
| mir-215 | ssc-mir-215 | ssc-mir-215 | 10 | 17031 | 10508 | 0 | 0.000411 | up | hsa-mir-215 |
| mir-222 | ssc-mir-222 | ssc-mir-222 | X | 247 | 151 | 0.000518 | 0.003882 | up | hsa-mir-222 |
| mir-26a | ssc-mir-26a | ssc-mir-26a | - | 8854 | 10360 | 0 | 0.000497 | up | hsa-mir-26a |
| mir-27b | ssc-mir-27b | ssc-mir-27b | 10 | 5154 | 5440 | 0 | 0.000682 | up | hsa-mir-27b |
| mir-29a | ssc-mir-29a | ssc-mir-29a | 18 | 199 | 234 | 0.002337 | 0.003713 | up | hsa-mir-29a |
| mir-29c | ssc-mir-29c | ssc-mir-29c | 9 | 230 | 265 | 0.00243 | 0.003458 | up | hsa-mir-29c |
| mir-30c | ssc-mir-30c | ssc-mir-30c | 6 | 938 | 649 | 7.00E-06 | 0.001865 | up | hsa-mir-30c |
| mir-30d | ssc-mir-30d | ssc-mir-30d | 4 | 5950 | 6439 | 0 | 0.000627 | up | hsa-mir-30d |
| mir-30e | ssc-mir-30e | ssc-mir-30e | 6 | 8291 | 8841 | 0 | 0.000528 | up | hsa-mir-30e |
| mir-320 | ssc-mir-320 | ssc-mir-320 | 14 | 877 | 640 | 0.000446 | 0.00191 | up | hsa-mir-320 |
| mir-339 | ssc-mir-339 | ssc-mir-339 | 3 | 552 | 316 | 0 | 0.002568 | up | hsa-mir-339 |
| mir-128–2 | ssc-mir-128 | ssc-mir-128–2 | 13 | 32 | 51 | 0.008046 | 0.008912 | up | hsa-mir-128 |
| mir-374a | ssc-mir-374a | ssc-mir-374a | X | 1051 | 657 | 0 | 0.001794 | up | hsa-mir-374a |
| mir-378 | ssc-mir-378 | ssc-mir-378–1  /ssc-mir-378–2 | 2/12 | 12575 | 14218 | 0 | 0.000417 | up | hsa-mir-378 |
| mir-423 | ssc-mir-423 | ssc-mir-423 | 12 | 1275 | 1909 | 0 | 0.001289 | up | hsa-mir-423 |
| mir-455 | ssc-mir-455 | ssc-mir-455 | 1 | 353 | 112 | 0 | 0.003575 | up | hsa-mir-455 |
| mir-7 | ssc-mir-7 | ssc-mir-7 | - | 222 | 459 | 0 | 0.00292 | up | hsa-mir-7 |
| mir-122 | ssc-mir-122 | ssc-mir-122 | 1 | 34 | 13 | 0.008563 | 0.012095 | up | hsa-mir-122 |
| mir-92a-1 | ssc-mir-92a | ssc-mir-92a-1 | 11 | 82 | 42 | 0.005108 | 0.007204 | up | hsa-mir-92a |
| mir-99a | ssc-mir-99a | ssc-mir-99a | 13 | 31 | 11 | 0.00815 | 0.012761 | up | hsa-mir-99a |
| let-7c | ssc-let-7c | ssc-let-7c | 13 | 219 | 242 | 0.013392 | 0.003591 | down | hsa-let-7c |
| mir-129 | ssc-mir-129 | ssc-mir-129 | 2 | 20 | 34 | 0.019848 | 0.011193 | down | hsa-mir-129 |
| mir-130b | ssc-mir-130b | ssc-mir-130b | 14 | 28 | 43 | 0.023441 | 0.009682 | down | hsa-mir-130b |
| mir-136 | ssc-mir-136 | ssc-mir-136 | 7 | 13 | 24 | 0.031382 | 0.013405 | down | hsa-mir-136 |
| mir-18 | ssc-mir-18 | ssc-mir-18 | 11 | 63 | 79 | 0.03527 | 0.006704 | down | hsa-mir-18 |
| mir-195 | ssc-mir-195 | ssc-mir-195 | 12 | 61 | 80 | 0.017795 | 0.00673 | down | hsa-mir-195 |
| mir-19b-1 | ssc-mir-19b | ssc-mir-19b-1 | 11 | 174 | 201 | 0.008166 | 0.004007 | down | hsa-mir-19b |
| mir-27a | ssc-mir-27a | ssc-mir-27a | 2 | 49 | 69 | 0.012534 | 0.007396 | down | hsa-mir-27a |
| mir-10b | ssc-mir-10b | ssc-mir-10b | 15 | 368 | 257 | 0.005498 | 0.003056 | down | hsa-mir-10b |
| mir-125b-1 | ssc-mir-125b | ssc-mir-125b-1 | 9 | 387 | 407 | 0.010433 | 0.002692 | down | hsa-mir-125b |
| mir-374b | ssc-mir-374b | ssc-mir-374b | X | 146 | 165 | 0.026744 | 0.004425 | down | hsa-mir-374b |
| mir-152 | ssc-mir-152 | ssc-mir-152 | 12 | 287 | 314 | 0.006858 | 0.00312 | down | hsa-mir-152 |
