# Supplementary material for: Analysis of Differential miRNA Expression in the Duodenum of Escherichia coli F18-Sensitive and -Resistant Weaned Piglets
Source: PLoS One. 2012 Aug 24;7(8):e43741. doi: 10.1371/journal.pone.0043741 (PMC3427155; doi:10.1371/journal.pone.0043741)
Supplement: Table S4 — Node attributes of all transcription factors. (DOC) [file pone.0043741.s008.doc]

**Table S**4. Node attributes of all transcription factors

| Transcription factor | Description | Degree |
| --- | --- | --- |
| SP1 | Sp1 transcription factor | 42 |
| NF1 | neurofibromin 1 | 39 |
| NFKB2 | nuclear factor NF-kappa-B p100 subunit-like | 5 |
| C-JUN | transcription factor AP-1 | 3 |
