# Supplementary material for: Analysis of Differential miRNA Expression in the Duodenum of Escherichia coli F18-Sensitive and -Resistant Weaned Piglets
Source: PLoS One. 2012 Aug 24;7(8):e43741. doi: 10.1371/journal.pone.0043741 (PMC3427155; doi:10.1371/journal.pone.0043741)
Supplement: Table S5 — miRNAs regulated by three different transcription factors. (DOC) [file pone.0043741.s009.doc]

**Table S**5 miRNAs regulated by three different transcription factors

| miRNA | Degree | Style |
| --- | --- | --- |
| ssc-mir-30e | 3 | up |
| ssc-mir-185 | 3 | up |
| ssc-mir-183 | 3 | up |
| ssc-mir-145 | 3 | up |
| ssc-mir-143 | 3 | up |
| ssc-mir-130a | 3 | up |
