# Supplementary material for: Analysis of Differential miRNA Expression in the Duodenum of Escherichia coli F18-Sensitive and -Resistant Weaned Piglets
Source: PLoS One. 2012 Aug 24;7(8):e43741. doi: 10.1371/journal.pone.0043741 (PMC3427155; doi:10.1371/journal.pone.0043741)
Supplement: Table S10 — The attribute relations between miRNAs in networks (degree >50) in Figure 1 . (DOC) [file pone.0043741.s014.doc]

**Table S10 The attribute relations between miRNAs in networks (degree >50) in Figure 1**

| miRNA | Degree | Style |
| --- | --- | --- |
| hsa-miR-181a | 59 | up |
| hsa-miR-181d | 59 | up |
| hsa-let-7a | 56 | up |
| hsa-let-7c | 56 | down |
| hsa-let-7e | 56 | up |
| hsa-let-7f | 56 | up |
| hsa-miR-15b | 52 | up |
| hsa-miR-195 | 52 | down |
