# Supplementary material for: Analysis of Differential miRNA Expression in the Duodenum of Escherichia coli F18-Sensitive and -Resistant Weaned Piglets
Source: PLoS One. 2012 Aug 24;7(8):e43741. doi: 10.1371/journal.pone.0043741 (PMC3427155; doi:10.1371/journal.pone.0043741)
Supplement: Table S11 — Relationships between GO categories (degree >25) in Figure 1 . (DOC) [file pone.0043741.s015.doc]

**Table S11 Relationships between GO categories (degree >25) in Supplementary Figure 1**

| **GO** | **Degree** |
| --- | --- |
| cell adhesion | 35 |
| positive regulation of transcription | 32 |
| BMP signaling pathway | 31 |
| response to stress | 31 |
| positive regulation of transcription from RNA polymerase II promoter | 30 |
| in utero embryonic development | 26 |
| positive regulation of apoptosis | 26 |
