# Supplementary material for: Analysis of Differential miRNA Expression in the Duodenum of Escherichia coli F18-Sensitive and -Resistant Weaned Piglets
Source: PLoS One. 2012 Aug 24;7(8):e43741. doi: 10.1371/journal.pone.0043741 (PMC3427155; doi:10.1371/journal.pone.0043741)
Supplement: Table S14 — Key miRNAs in network (degree >15). (DOC) [file pone.0043741.s018.doc]

**Table S14 Key miRNAs in network of Figure 2(degree >15)**

| miRNA | style | Degree |
| --- | --- | --- |
| hsa-miR-15b | up | 19 |
| hsa-miR-195 | down | 19 |
| hsa-miR-19b | down | 18 |
| hsa-let-7a | up | 17 |
| hsa-let-7c | down | 17 |
| hsa-let-7e | up | 17 |
| hsa-let-7f | up | 17 |
| hsa-miR-130a | up | 17 |
| hsa-miR-130b | down | 17 |
| hsa-miR-148a | up | 16 |
| hsa-miR-148b | up | 16 |
| hsa-miR-152 | down | 16 |
